# Pathogenic prion structures at high resolution

**DOI:** 10.1371/journal.ppat.1010594

**Published:** 2022-06-30

**Authors:** Byron Caughey, Heidi G. Standke, Efrosini Artikis, Forrest Hoyt, Allison Kraus

**Affiliations:** 1 Rocky Mountain Laboratories, National Institute of Allergy and Infectious Diseases, NIH, Hamilton, Montana, United States of America; 2 Department of Pathology and Cleveland Center for Membrane and Structural Biology, Case Western Reserve University School of Medicine, Cleveland, Ohio, United States of America; 3 Research Technologies Branch, Rocky Mountain Laboratories, National Institute of Allergy and Infectious Diseases, National Institutes of Health, Hamilton, Montana, United States of America; Stanford University, UNITED STATES

Numerous proteins are known to form ordered, self-propagating aggregates as underlying causes of neurodegeneration in proteinopathies such as Alzheimer’s, Parkinson’s, and prion diseases [1,2]. The prion-like properties of such aggregates can promote spreading within and, sometimes, between hosts as pathogens. Prions are protein-based transmissible agents that, unlike other types of pathogens, do not carry a specific nucleic acid genome. Classical prions are formed from the hosts’ native prion protein (PrP^C^) and have long been known to be highly transmissible in many mammalian species (reviewed in [2,3]). Other more common proteinopathies have also been shown to be transmissible from humans to experimental, and usually transgenic “humanized,” animals, but the extent to which any such transmission occurs between humans remains under investigation and debate [4]. Extensive comparisons of brain-derived versus synthetic PrP aggregates with in vitro self-propagating (seeding) activities have shown that conformational differences can profoundly affect their in vivo transmissibilities and pathogenicities, or lack thereof (e.g., [2,5]). For example, whereas approximately 10^‒15^ g of brain-derived prions can be lethal, inoculation of a >10^9^-fold larger amount of synthetic recombinant PrP fibrils can be innocuous [2]. Thus, in evaluating and understanding the risks posed by various self-replicative protein assemblies, it is crucial to consider conformational details. Fortunately, although the structures of prions and other proteopathic aggregates eluded high-resolution determination for decades, recent applications of cryogenic electron microscopy (cryo-EM) have begun to reveal these structures (e.g., [6–11]). Within the last year and a half, the first 3 near-atomic resolution cryo-EM structures of highly pathogenic brain-derived prions have been reported [6–10]. Here, we highlight how these new prion structures have begun to address fundamental, long-standing questions in prion biology.

## What structures allow prions to propagate as deadly infectious pathogens?

**Fig 1:**
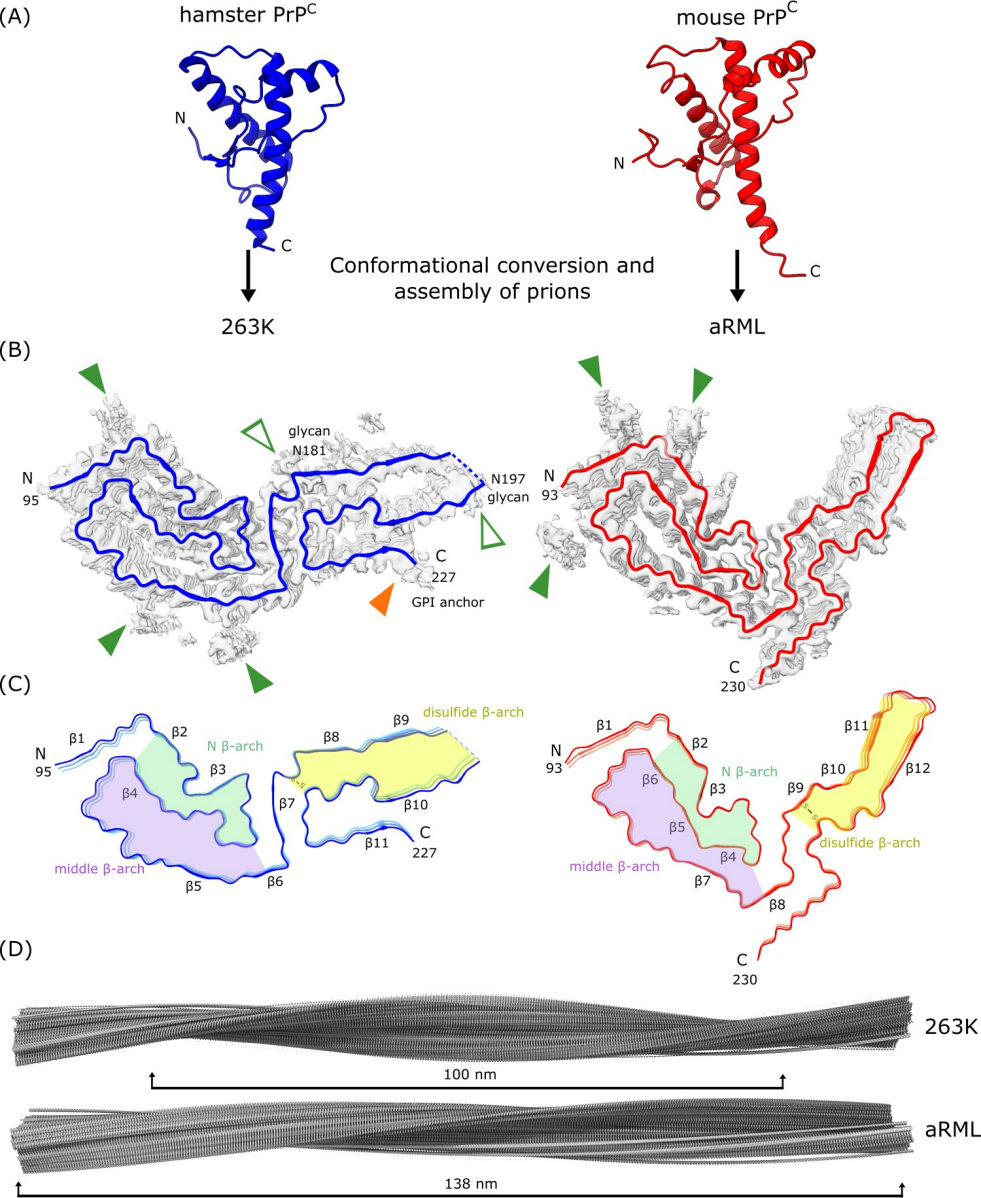
Two prion strains are amyloid fibrils with distinct templating surfaces on the ends where incoming monomers are refolded. (A) PrP^C^ molecules (disordered N-terminal residues not shown) undergo conversion and assembly into the prion forms shown here for hamster 263K (blue, PDB: 7LNA, [6,7]) and mouse aRML (red, PDB: 7TD6, [8,9]) prions. PDB accession codes 1B10 and 1XYZ were used for hamster and mouse PrP^C^, respectively. (B) Polypeptide backbones of the fibril cross-sections (colored) as they fit within the cryo-EM density maps (gray). Open arrowheads: densities outside the polypeptide backbone that coincide with attachment sites of glycans (green) or glycolipid anchors (orange). Closed green arrowheads: densities of unknown origin outside the amyloid cores, often adjacent to cationic residues. (C) Similar shared β-arch motifs/topologies distinguished by color. Variations within these topologies and other features, including distinct orientations of the C-terminal tails, distinguish these strains. (D) Extended lateral views of fibril density maps, with brackets indicating the relative cross-over distances. cryo-EM, cryogenic electron microscopy; PrP, prion protein.

**Fig 2:**
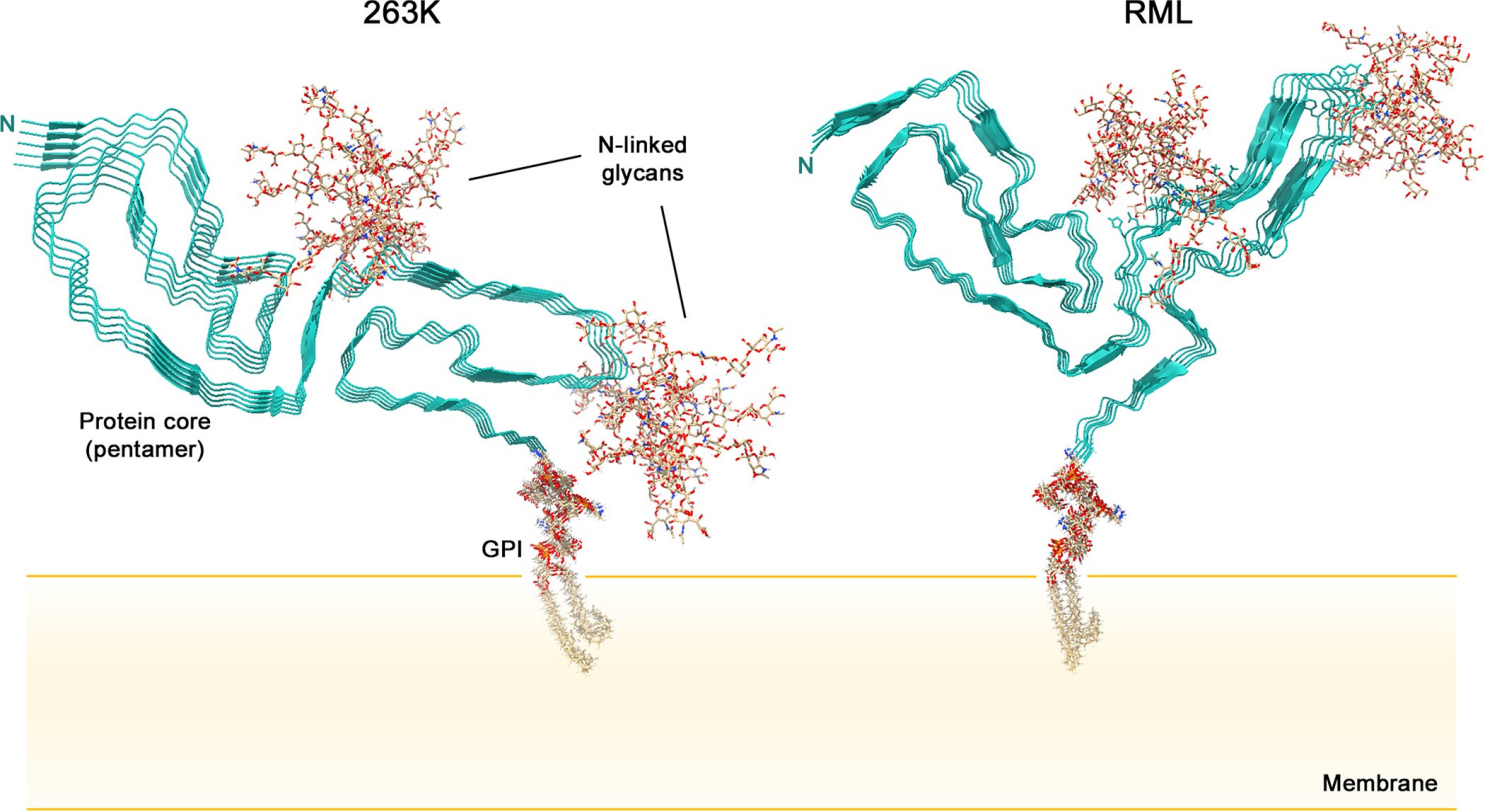
Cross-sectional depictions of membrane-bound 263K and RML prions. For simplicity, only short pentameric segments of the fibrils are shown. The natural glycans and GPI moieties are much more heterogeneous than those shown. The RML structure shown was assembled using the aRML pdb coordinates (PDB: 7TD6) because the wtRML pdb coordinates were not yet publically available. Although the aRML and wtRML cores are similar overall, there are detailed features of the wtRML core structure that differ from that depicted. See [10]. GPI, glycophosphatidylinositol.

Although divergent ultrastructures have been observed in ex vivo preparations of infectious prions, the high-resolution prion structures that are available so far are amyloid fibrils (**Fig 1**) [6–10]. These structures are of the hamster scrapie strain 263K [6,7] and the mouse wild type (wt) [10] and glycophosphatidylinositol (GPI)-anchorless (a) [8,9] RML scrapie strains. As for most amyloids, these prion fibrils have highly ordered core structures with PrP molecules stacked parallel and in register such that each monomer provides 1 rung along the fibril axis and any given amino acid residue in 1 monomer is aligned axially with the analogous residues of other monomers. Whereas most amyloid fibrils have 2 or more protofilaments, these prion fibrils consist primarily of 1 protofilament, the cross-section of which is spanned by a single monomer (**Fig 1B** and **1C**). To accommodate up to 137 residues within the ordered core cross-sections, the PrP polypeptide backbone of each rung folds back on itself in a serpentine fashion, forming a series of β-sheet sections called parallel in-register intermolecular β-sheets (PIRIBS) that are stabilized by hydrogen bonds between backbone carbonyls and amides. The β-sheets are interspersed with loops or turns. The structurally diverse N-linked glycans, and GPI anchors, if present, are not well resolved in these cryo-EM analyses and are presumed to decorate the periphery of the fibril (**Fig 2**). Also poorly resolved are the less ordered, primarily N-terminal amino acid residues.

The ends of the prion fibrils provide templates for the addition of new monomers as the fibrils grow [6–10]. Inherent in these structures is the fact that the templating surfaces at opposite ends of a given fibril are not equivalent, raising the possibility of different elongation kinetics at these sites. Given that the normal PrP^C^ precursor of the infectious prion isoform (generically called PrP^Sc^ for PrP-scrapie) is monomeric with predominantly disordered and α-helical secondary structural domains (**Fig 1A**), a wholesale refolding occurs upon recruitment of new monomers into fibrils [6,7]. Much remains to be determined about the steps involved, but among the key events are the peeling of the loop containing helix 1 away from helices 2 and 3 of PrP^C^ [12] and stretching of the helices into extended strands within the prion fibril core [6,7]. A clearer understanding of this conversion mechanism should aid in the rational design of therapeutic strategies aimed at limiting prion propagation.

## What is the structural basis of prion strain variation?

Comparison of the 263K and RML prion structures gives initial insights into prion strain variation [6–10]. These rodent-adapted scrapie strains share structural motifs including N (N-proximal), middle, and disulfide β-arches, and a steric zipper that glues the N-terminal residues of the core to the head of the middle β-arch (**Fig 1C**). However, otherwise, there are striking differences in the detailed conformations of these and other features, as well as the overall fibril morphologies (**Fig 1B–1D**). Particularly notable are the different orientations of the C-terminal residues linked to the GPI anchor in the 263K and wtRML prions [6–10]. In 263K, these residues flank the disulfide β-arch but extend in the opposite direction in the RML structures. In addition, the amino acid sequences within the cores of 263K and RML prions differ at 8 positions in their respective hamster and mouse PrP molecules. The extent to which the conformational differences between 263K and RML prions are dictated by sequence differences versus conformational templating remains to be determined. Nonetheless, although only 3 high-resolution infectious prion structures are currently available, we can already surmise that major determinants of prion strains are conformational variations in common motifs and other structural elements [8,9]. However, there are more than 30 different types of PrP-based prions in mammals including various strains, sequences, and combinations thereof. Thus, much remains to be done to explain the full range of prion structures and how those structures underpin strain-specific disease phenotypes.

## How might prion structures explain prion transmission (and species) barriers?

Many lines of evidence indicate that a primary factor controlling the transmissibility of prions between hosts of different genotypes is sequence compatibility between the infecting PrP^Sc^ and the host’s PrP^C^ (e.g., [7] and references therein). While some sequence differences have little impact, others can result in high, and seemingly insurmountable, barriers to transmission. The structural bases for these effects remain poorly understood, but the new high-resolution prion structures provide initial insights into potential transmission barrier mechanisms [7]. Each of these PIRIBS-based structures has tightly packed regions in which the substitution of a heterologous residue might cause unfavorable steric clashes, electrostatic interactions, changes in hydrogen bonding, or other perturbations that could slow or prevent full conformational conversion of incoming monomers. On the other hand, other areas of these fibril cores are less packed or otherwise more tolerant of heterologous residues. Even if heterologous conversion can occur sustainably in the new host, an altered or partial conversion product might be less pathogenic or more easily cleared than the original prion strain in its original host, and therefore, less causative of clinical disease within the natural lifespan of the new host.

## How might GPI anchors and N-linked glycans affect prion structure and interactions with tissue environments?

GPI anchors and N-linked glycans are prominent features of wild-type prions that are unusual among the many pathologic protein amyloids of mammals. Although these post-translational modifications are not required for prion infectivity or pathogenicity, they can profoundly affect the routes of prion spreading within the host [13] and the resulting clinical and neuropathological phenotypes of disease [14]. In the 263K and wtRML prion structures, the anchors and glycans can be inferred to project outward from the fibril cores (**Fig 2**) [6,7,10]. The resulting array of GPI anchors along the fibril axis would likely interact with cellular membranes, perhaps causing distorted membrane lesions that are indicative of prion-infected brain tissue [6,7]. Such lesions might in turn mediate disruptions of cellular functions such as membrane trafficking, signaling, and homeostasis. The attached membranes, as well as N-linked glycans, should obscure at least the C-terminal cores of the prion fibrils in a manner that would limit interactions with other macromolecules. Disease progression is often faster in wild-type versus anchorless PrP transgenic mice [15], suggesting that GPI-mediated membrane interactions contribute significantly to the neuropathogenic process. However, it is important to emphasize that both wild-type and GPI-anchorless prions, the latter of which are also deficient in N-linked glycans, can be lethal to the host [14,15]. Indeed, after multiple serial passes of scrapie strains in anchorless PrP transgenic mice, survival times can become as short as those in wild-type mice [16,17]. Thus, there must be both GPI-dependent and -independent mechanisms by which prions disrupt brain function.

## What are structural determinants of prion transmissibility and pathogenicity?

Structural features correlating with infectivity are already suggested by comparisons of cryo-EM structures of brain-derived infectious prions [6–10] to presumably much less, if at all, infectious synthetic PrP fibrils that also all have PIRIBS-based cores [18–22]. Assessments of the infectivity of latter synthetic structures have not been reported, but most recombinant PrP preparations with similarly small protease-resistant cores have lacked pathogenicity. However, we note that one of these high-resolution cryo-EM structures is of fibrils of human PrP 23–144, and a fibrillar preparation of the analogous murine fragment induces prion disease in PrP-overexpressing transgenic mice [23], albeit with long incubation periods suggestive of low titer. In any case, whereas the high-titered infectious PrP fibrils (approximately 10^8^ to 10^10^ 50% lethal doses per mg) have large ordered amyloid core structures with an N-proximal steric zipper as well as N, middle, and disulfide β-arches [6–10], the available synthetic fibril structures have only either an N or a disulfide β-arch without the other major features. Importantly, however, even seemingly subtle differences in the size of PrP fibril cores (e.g., as little as approximately 1 kDa [5,24]) have correlated with large differences in infectivity [2]. Thus, much remains to be learned about the structure–function relationships that discriminate lethal from innocuous assemblies of PrP molecules.
